# Free-water imaging of the cholinergic basal forebrain and pedunculopontine nucleus in Parkinson’s disease

**DOI:** 10.1093/brain/awac127

**Published:** 2022-04-29

**Authors:** Nicola J Ray, Rachael A Lawson, Sarah L Martin, Hilmar P Sigurdsson, Joanna Wilson, Brook Galna, Sue Lord, Lisa Alcock, Gordon W Duncan, Tien K Khoo, John T O’Brien, David J Burn, John-Paul Taylor, River C Rea, Maurizio Bergamino, Lynn Rochester, Alison J Yarnall

**Affiliations:** Health, Psychology and Communities Research Centre, Department of Psychology, Manchester Metropolitan University, Manchester, UK; Translational and Clinical Research Institute, Newcastle University, Newcastle upon Tyne, UK; Health, Psychology and Communities Research Centre, Department of Psychology, Manchester Metropolitan University, Manchester, UK; Translational and Clinical Research Institute, Newcastle University, Newcastle upon Tyne, UK; Translational and Clinical Research Institute, Newcastle University, Newcastle upon Tyne, UK; Translational and Clinical Research Institute, Newcastle University, Newcastle upon Tyne, UK; Health Futures Institute, Murdoch University, Perth, Australia; Auckland University of Technology, Auckland, New Zealand; Translational and Clinical Research Institute, Newcastle University, Newcastle upon Tyne, UK; Centre for Clinical Brain Sciences, University of Edinburgh, Edinburgh, UK; NHS Lothian, Edinburgh, UK; School of Medicine & Dentistry, Menzies Health Institute Queensland, Griffith University, Queensland, Australia; School of Medicine, University of Wollongong, Wollongong, New South Wales, Australia; Department of Psychiatry, University of Cambridge, Cambridge, UK; Population Health Sciences Institute, Newcastle University, Newcastle upon Tyne, UK; Translational and Clinical Research Institute, Newcastle University, Newcastle upon Tyne, UK; Health, Psychology and Communities Research Centre, Department of Psychology, Manchester Metropolitan University, Manchester, UK; Barrow Neurological Institute, Neuroimaging Research, Phoenix, AZ, USA; Translational and Clinical Research Institute, Newcastle University, Newcastle upon Tyne, UK; The Newcastle upon Tyne NHS Foundation Trust, Newcastle upon Tyne, UK; Translational and Clinical Research Institute, Newcastle University, Newcastle upon Tyne, UK; The Newcastle upon Tyne NHS Foundation Trust, Newcastle upon Tyne, UK

**Keywords:** free-water imaging, cholinergic system, pedunculopontine nucleus, nucleus basalis of Meynert, cognitive decline

## Abstract

Free-water imaging can predict and monitor dopamine system degeneration in people with Parkinson’s disease. It can also enhance the sensitivity of traditional diffusion tensor imaging (DTI) metrics for indexing neurodegeneration. However, these tools are yet to be applied to investigate cholinergic system degeneration in Parkinson’s disease, which involves both the pedunculopontine nucleus and cholinergic basal forebrain.

Free-water imaging, free-water-corrected DTI and volumetry were used to extract structural metrics from the cholinergic basal forebrain and pedunculopontine nucleus in 99 people with Parkinson’s disease and 46 age-matched controls. Cognitive ability was tracked over 4.5 years.

Pearson’s partial correlations revealed that free-water-corrected DTI metrics in the pedunculopontine nucleus were associated with performance on cognitive tasks that required participants to make rapid choices (behavioural flexibility). Volumetric, free-water content and DTI metrics in the cholinergic basal forebrain were elevated in a sub-group of people with Parkinson’s disease with evidence of cognitive impairment, and linear mixed modelling revealed that these metrics were differently associated with current and future changes to cognition.

Free water and free-water-corrected DTI can index cholinergic degeneration that could enable stratification of patients in clinical trials of cholinergic interventions for cognitive decline. In addition, degeneration of the pedunculopontine nucleus impairs behavioural flexibility in Parkinson’s disease, which may explain this region’s role in increased risk of falls.

## Teaser

Ray *et al*. demonstrate that free-water imaging can be used to measure degeneration of the pedunculopontine nucleus in Parkinson’s disease. Degeneration of this region is associated with impaired behavioural flexibility, confirming findings from the animal literature.

## Introduction

Degeneration of the dopaminergic substantia nigra is a hallmark of Parkinson’s disease (PD). Cholinergic cells of the basal forebrain (cBF) and pedunculopontine nucleus (PPN) are also implicated,^1,2^ but their roles in PD progression and symptomology remain unclear. It is important that we understand the spatiotemporal patterns of cBF and PPN degeneration, and their relationship to symptoms, if we are to make rational decisions about how treatments that target the cholinergic system are developed and utilized.

*In vivo* structural imaging studies imply that degeneration of the cBF in people with PD is associated with the development of cognitive impairments.^3–6^ Given the heterogenous involvement of the cholinergic deficit in PD, these metrics may be useful to identify people at risk of more serious cognitive decline. On the other hand, PPN degeneration has been implicated in PD axial motor symptoms such as posture and gait deficits.^7–9^ However, the traditional view of the PPN as a purely motor structure is under challenge.^10–13^ Current thinking suggests the PPN is critical for behavioural flexibility (adapting actions based on changing environmental contingencies).^11^

Diffusion tensor imaging (DTI) has been used to index degeneration in subcortical grey matter structures in people with PD via changes in fractional anisotropy (FA) and diffusivity.^14^ In particular, mean diffusivity (MD) and axial diffusivity (AD) have been used to investigate the impact of degeneration in the cholinergic nuclei.^4,6,8^ However, these traditional DTI indices assume a single-tissue compartment per voxel, thereby conflating the representation of free water (FW) and tissue. FW is present as CSF and also accumulates extracellularly due to neuroinflammation.^15^ This confound may hinder the sensitivity of DTI metrics in cholinergic nuclei from identifying people with evidence of cholinergic degeneration who may be candidates for current and future cholinergic therapy.

FW imaging can determine FW content (fractional volume, FWf) and correct for this when estimating tissue microstructures. In PD, FW imaging of the substantia nigra is emerging as a promising biomarker for distinguishing people with PD from healthy individuals,^16^ and for monitoring disease progression.^16–19^ Whether this imaging technique can also be used to identify people with PD with evidence of degeneration in the cBF and PPN is not currently known. Yet, with the ongoing development of promising therapeutics that target the cholinergic system,^20,21^ an objective cholinergic biomarker is urgently needed. We therefore sought to evaluate (i) whether FW imaging in the cBF and PPN can distinguish people with PD at early disease stages from controls; (ii) if these metrics can identify people with PD with evidence of cognitive impairment or predict the emergence of this over time; and (iii) if FW and FW-corrected DTI metrics can help us to understand the contributions of cBF and PPN degeneration to different cognitive symptoms in PD.

## Materials and methods

### Participants

Participants with idiopathic PD and age-matched controls were recruited to the ICICLE (Incidence of Cognitive Impairment in Cohorts with Longitudinal Evaluation)—Parkinson’s disease study, with optional additional recruitment into the collaborative ICICLE-GAIT study. Recruitment was conducted between June 2009 and December 2011.^22,23^ Exclusion criteria included more advanced cognitive impairment [Mini-Mental State Examination (MMSE) ≤24], PD dementia at baseline,^24^ diagnosis of Parkinsonian disorders other than PD and poor command of the English language. Clinical and cognitive assessments were completed at baseline and three subsequent follow-up sessions: 18 months, 36 months and 54 months. MRI was completed at baseline. Idiopathic PD was diagnosed according to the Queen Square Brain Bank criteria,^25^ and diagnoses were reviewed at each assessment to reduce misdiagnosis risk. Participants were tested ‘on’ dopaminergic medication for all assessments.

Participants within the current analysis were those selected in Wilson *et al*.^26^ from the ICICLE-GAIT study who also had a DTI scan at baseline. This selection allows us to interpret our findings in the context of outcomes from Wilson *et al*.,^26^ and though not in scope of the current paper, to extend our analyis to investigate progressive changes to gait. A total of 99 people with PD and 46 controls were included in the current analysis. Following MRI quality control (see ‘MRI pre-processing’ section below), two people with PD and six control participants and were excluded, leaving 97 people with PD and 40 controls. The study was approved by the Newcastle and North Tyneside Research and Ethics Committee (REC no. 08/H0906/147).

### Clinical assessments

Age, sex, years of education and Movement Disorder Society Unified Parkinson’s Rating Scale (MDS-UPDRS III) scores were recorded. Global cognition was assessed through the MMSE and Montreal Cognitive Assessment (MoCA). Levodopa equivalent daily dose (LEDD) was calculated using methods described by Tomlinson *et al*.^26,27^ Participants also completed a battery of neuropsychological tests (see Lawson *et al*.^28^) Executive function was assessed using the One Touch Stockings (OTS) test from the Cambridge Neuropsychological Test Automated Battery (CANTAB),^29^ phonemic fluency (composite score of number of words generated in 60s beginning with the letters F, A and S) and semantic fluency (number of animals generated in 90s). Visual memory was assessed using the Pattern Recognition Memory (PRM), Spatial Recognition Memory (SRM) and Paired Associate Learning (PAL) tests from CANTAB.^29^ Attention was assessed using the Cognitive Drug Research (CDR) battery,^30^ including mean reaction time in milliseconds of simple reaction time (SRT), choice reaction time (CRT) and digit vigilance (DV); accuracy of CRT and DV were measured as percentage correct. Mean response times of SRT, CRT and DV were summed to produce a power of attention (PoA) score; fluctuating attention was measured using the coefficient of variance of PoA reaction variability (PoA CoV). Cognitive reaction time was the mean difference in in reaction time between SRT and CRT. Spatial working memory was assessed using the Spatial Working Memory (SWM) test, also from the CDR battery.

#### Cognitive status

At baseline, people with PD with evidence of cognitive impairment were identified with MoCA (MoCA < 26 indicates potential mild cognitive impairment), while those with scores greater than 25 have normal cognition.^31^

### MRI

#### MRI acquisition

MRI acquisition was performed using a 3 T Philips Intera Achieva scanner. A magnetization-prepared rapid acquisition gradient echo (MP-RAGE) T_1_-weighted sequence produced high-resolution structural images with the following parameters: repetition time = 9.6 ms, echo time = 4.6 ms, flip angle = 8°, SENSE factor = 2, field of view = 240 × 240 mm, voxel size = 1.5 × 1.5 × 1.5 mm^3^, acquisition time = 4 min, 150 sagittal slices (slice thickness = 1.2 mm). DTI acquisitions were based on a 2D diffusion-weighted, spin-echo, echo planar imaging sequence with 59 slices: repetition time = 6100 ms; echo time = 70 ms; flip angle = 90°; voxel size = 2.1 × 2.1 mm; slice thickness = 2.1 mm; field of view = 270 × 270 mm. Diffusion weighting was performed in 64 directions (diffusion b = 1000 s/mm^2^) and in six acquisitions without diffusion weighting (B0).

#### Image pre-processing

T_1_-images were first segmented into separate grey, white and CSF tissue compartments for DARTEL initialization, implemented in SPM12 (https://www.fil.ion.ucl.ac.uk/spm/software/spm12/). DARTEL performs a diffeomorphic algorithm for intersubject registration, producing individual flow field maps (which parameterize the deformation of the images) as well as average grey and white matter templates.^32^ Pre-processed grey matter maps were visually inspected for segmentation and registration accuracy, resulting in removal of one control participant.

For the diffusion images, after brain extraction, eddy current-induced distortion and subject movements were corrected using the Eddy FSL toolbox. Participants were removed if they had more than 2 mm absolute mean displacement, resulting in the removal of five further controls and one PD participant. FW corrected fractional anisotropy (cFA), mean diffusivity (cMD), axial diffusivity (cAD) and FW images were created by fitting the bi-tensor model described by Pasternak *et al*.^39^ to the raw diffusion data using custom MATLAB scripts. To align these images with T_1_-anatomical images, the B0 scan was extracted and affine registered to the T_1_ image using antsRegistrationSyn.sh [Advanced Neuroimaging Tools (ANTs)].^33^

#### Regions of interest: cBF and PPN stereotactic maps

Stereotactic mapping of cBF nuclei was used to create the cBF map, as described by Kilimann *et al*.^34^ Briefly, the map was derived from a brain specimen of a 56-year-old male who died from myocardial infarction. This underwent histological preparation and post-mortem MRI scans, both *in situ* and after the brain was dehydrated for histological preparation. Mesulam’s nomenclature^35^ was followed to identify cholinergic nuclei on digital pictures of stained brain slices; these were manually transferred into corresponding MR slices of the dehydrated brain. The MRI scan of the dehydrated brain was transformed into the space of the post-mortem in situ scan, using an initial 12-parameter affine transformation followed by a high-dimensional nonlinear registration between the two scans.^36^ This was transferred to Montreal Neurological Institute (MNI) standard space to enable use of the high-dimensional DARTEL (Diffeomorphic Anatomic Registration using Exponentiated Lie algebra) registration method.^32^ The final stereotactic map distinguishes different cBF subdivisions, including cholinergic cell clusters corresponding to the medial septum, vertical and horizontal limb of the diagonal band, and the nucleus basalis of Meynert. Following previous PD literature using this cBF mask,^34^ regions of interest (ROIs) selected for analysis were: (i) a combination of the medial septum (Ch1) and vertical limb of the diagonal band (Ch2); and (ii) the nucleus basalis of Meynert (Ch4).

Stereotactic mapping of the PPN was also used to create the PPN mask, as described by Alho *et al*.^37^ Briefly, post-mortem MRI was performed on the brain of a 66-year-old woman without parkinsonism or cognitive decline. Following autopsy, the brain was fixed, dehydrated, serially sectioned and Nissl stained. Light and darkfield microscopy was used to enhance contrast and perform the segmentation of the nuclear boundaries of the PPN, creating a mask of the entire PPN region. Following digitization, the images were 3D registered with the post-mortem MRI and the PPN mask transformed to MNI space via transforms generated following normalization of the post-mortem MRI to MNI space.

#### Extraction of volumetric, FW and FW-corrected diffusivity metrics from ROIs

Previous research has evaluated whether volumes of the cBF in people with PD are associated with cognitive impairments.^3–6^ We also extracted this volumetric information from the cBF as in Wilson *et al*.,^26^ which also used the ICICLE-GAIT dataset. Briefly, this involved spatial normalization to the MNI-space ICBM152 brain, extraction of volumes from within the MNI-space cBF ROIs, and subsequent normalization to total intracranial volume (TIV) via ANCOVA. However, as described previously,^8^ volumetric analysis is not possible using the techniques used here for the PPN, given its brainstem location.

For FW and FW-corrected metrics, we first transformed MNI-space ROI images (described in ‘Regions of interest: cBF and PPN stereotactic maps’ section) to native space as follows: Participant’s T_1_ images were affine registered to their B0 image (extracted from the DWI) using antsRegistrationSyn.sh ANTs.^33^ The T_1_ image was also affine registered to the MNI-space ICBM152 brain.^38^ The resulting inverse transform from the latter was used to transform the MNI-space PPN and cBF ROI maps to T_1_ space, and the transform from the former was used to transform into B0 space. All warps of the ROI maps used nearest-neighbour interpolation. All PPN and cBF maps in native space were inspected for accuracy, and one participant with PD was removed due to misalignment.

To ensure only grey matter voxels were included in ROIs, voxels within the ROI maps were conditioned on FA, following Schulz *et al.*^6^ For the PPN, which has white matter tracts from the brain stem coursing through it, voxels with FA greater than 0.47 (following values reported in Alho *et al*.,^37^ i.e. mean ± 1 SD) were removed. In the cBF, which should not have the same degree of white matter contamination, voxels with FA values greater than 0.3 were removed. In addition, for the cBF, any voxels not segmented as grey matter during T1 image processing (described above, i.e. not present in the grey matter segmented image) were also removed from the ROIs. Mean FWf, cMD and cAD were calculated from the remaining voxels within each ROI.

In summary, there were four metrics from each of the cBF ROIs: volume, FWf, cMD and cAD; and three metrics from the PPN: FWf, cMD and cAD.

### Statistical analysis

Analyses were conducted using SPSS (IBM Corp. V.24, USA) and R software (R Foundation for Statistical Computing, V3.5.2, Austria).

#### Data cleaning

The distribution of continuous variables was tested for normality with Kolmogorov–Smirnov tests and boxplot and histogram inspections. Some of the imaging metrics deviated from a normal distribution, tending to be left skewed, which is not easily ‘normalized’ with transformation. Given the large sample size (for which normality is a less important assumption) and the analytical approach (described below), we opted to clean the data of extreme outliers and proceed with parametric testing. As such, all data (including clinical and imaging) were cleaned of extreme outliers (3× greater than interquartile range) prior to analysis. At baseline, this resulted in the removal of two data points for simple reaction time and one data point for choice reaction time. For the imaging metrics, 12 data points in total were removed across FWf, cAD and cMD in PPN, Ch1-2, Ch4 and whole-brain grey matter.

#### Baseline diffusivity metrics and cognitive scores

One-way ANOVA with post hoc Student’s *t*-tests assessed differences in baseline cognitive scores and structural metrics in the cBF and PPN between controls and people with PD. Given previous reports that showed differences in cBF structural metrics only when comparing people with PD with/without cognitive impairment,^3,6^ people with PD were then further separated into those with and without evidence of early cognitive impairment (MoCA < 26 and MoCA > 25, respectively^31^). Comparisons that were significant at *P* < 0.05 after FDR correction (see below) were further evaluated with correction for age, sex and whole-brain structural metrics using ANCOVA.

Pearson’s bivariate correlations examined within-group relationships between baseline cognitive scores and cBF and PPN structural metrics. All bivariate correlations significant at *P* < 0.05 (FDR corrected) were further evaluated using partial correlations (controlling for age, sex and whole-brain FW or FW-corrected diffusivity).

#### Baseline diffusivity metrics and cognitive changes at follow up

Linear mixed-effects models (LMM; R, ‘lme4’^39^ and ‘lmerTest’^40^) separately modelled change in each cognitive test over the 54-month follow-up period. LMM can effectively handle the hierarchical nature of longitudinal, repeated-measures data, with missing data accounted for using maximum likelihood estimation, allowing us to take advantage of the full 54-month follow-up period without any case-wise deletion due to missing data points. Random slope models gave each participant a unique intercept and slope, allowing for correlation between intercept and slope. Baseline age, sex, cognitive scores and whole-brain diffusivity were included as fixed effects, and model fit was assessed by likelihood ratio tests. The interaction between structural metrics and time were additionally modelled to determine if these metrics were associated with cognitive changes over the follow-up period (e.g. time × cAD).

For figures illustrating the LMM outcomes, we modelled rate-of-change in cognitive scores using the beta parameters estimated by the model. This can be thought of as an estimate of the change likely to occur between a visit and its subsequent follow-up 18-months later, given the values of the predictors for each participant.

#### Multiple comparisons

In general, our statistical approach is to perform *t*-tests and bivariate correlations first and only take significant results into ANCOVAs and partial correlations or regression. This is intended to transparently report the data (i.e. so it is clear that our outcomes do not depend on the addition of particular covariates). Correction for multiple comparisons is applied at the level of the *t*-tests and bivariate correlations via false discovery rate (FDR) correction. The same correction is applied to the LMM outcomes for the longitudinal data. FDR is applied at least for the number of diffusivity metrics compared within an ROI (for example, in the PPN, we have corrected for the fact that fWF, cAD and cMD are tested).

Volumes of the cBF have been consistently shown to be associated with cognitive impairment.^3–6^ We therefore did not include *P*-values related to volumetry in the FDR corrections. For clarity, in the results section and in table legends we indicate when comparisons have been corrected for.

### Data availability

Requests to use the ICICLE-gait dataset should be made to the PIs on that project (author L.R.). For the free-water and DTI metrics, readers are directed to author N.R.

## Results

Following exclusions due to quality control of MR images, 96 people with PD and 40 control participants were included in the current analysis. Of these, at 18 months, 90 people with PD and 37 control participants were available. At 36 months, 78 people with PD and 31 control participants were available, and at 54 months, 66 people with PD and 24 control participants were available. A number of factors led to this attrition, including participants withdrawing from the study, being lost to follow up, or due to death. None of the participants initiated deep brain stimulation treatment within the timeframe of the study. NB: for some participants, cognitive data are missing at 54 months due to a protocol change, rather than due to attrition. Comparisons between demographic and clinical scores for the sample included here at baseline are reported in Table 1.

**Table 1:** Baseline clinical data in controls and people with PD

	Control, *n* = 40 (female = 15)		PwP, *n* = 96 (female = 33)	
	Mean	SD	Mean	SD
Age, years	66.69	7.60	65.66	10.65
Education, years	14.0	3.80	13.5	4.0
MoCA	27.8	1.81	25.33	3.53
Disease duration, months	—	—	6.46	4.84
MDS-UPDRS (Part III)			25.12	10.12
LEDD, mg/day	—	—	169.76	127.21

PwP = people with PD.

### Do structural metrics in cholinergic nuclei at baseline distinguish people with PD from controls?

None of the structural metrics were significantly elevated in people with PD as a whole compared with controls (Table 1).

### Are structural metrics in cholinergic nuclei associated with cognition at baseline?

**Figure 1:**
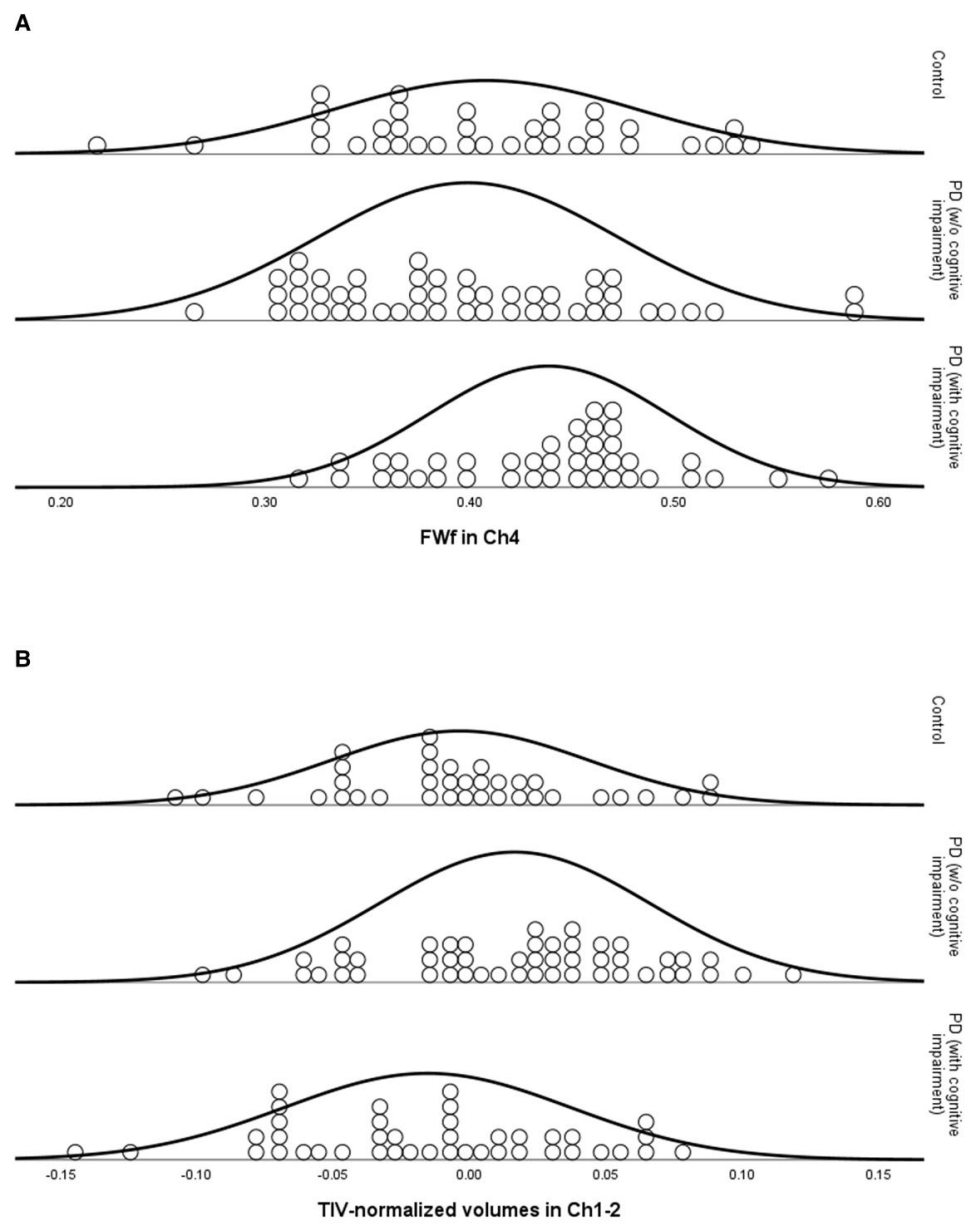
**Dot plots of structural metrics in cBF by Group:** (**A**) Circles represent FWf in the Ch4 region of basal forebrain, (**B**) circles represent total intracranial volume-normalized volumes of the Ch1-2 region of basal forebrain. Groups are in controls, people with PD with MoCA scores > 25 [PwP (NC)] and people with PD with MoCA scores < 26 [PwP (CI)]. Normal distribution lines are overlayed.

One-way ANOVAs with *post hoc t*-tests revealed that people with PD with cognitive impairment at baseline had increased FWf in Ch4 compared to controls and people with PD without cognitive impairment (FDR corrected; Fig. 1A). cAD in this region was also elevated in people with PD with (compared to without) cognitive impairment (statistics reported in Table 1), and these differences survived control for age, gender and whole-brain structural metrics (FWf: *F* = 4.93, *P* = 0.03; cAD: *F* = 6.96, *P* = 0.01).

One-way ANOVA and *post hoc t*-tests revealed that volumes in Ch1-2 were larger in people with PD without cognitive impairment compared to both controls and people with PD with evidence of cognitive impairment. However, these outcomes did not survive control for age, sex and whole-brain grey matter (Fig. 1B, see Table 2 for statistics).

**Table 2:** Baseline clinical data and structural metrics in Ch1-2, Ch4 and PPN

			PwP (*n* = 90)					
	Controls (*n* = 40)		MoCA > 25 (*n* = 49)		MoCA < 26 (*n* = 41)			
		SD	Mean	SD	Mean	SD	Statistic	*P*-value
Ch1-2 (mm^3^)	−0.004	0.047	0.016^a,b^	0.050	−0.015^b^	0.052	*F* = 4.75, *t* = 1.88^a^, *t* = 2.97^b^	*P* = 0.01 (uncorrected), *P* = 0.04, *P* = 0.01
Ch1-2 FWf	0.486	0.114	0.459	0.116	0.514	0.124	*F* = 2.35	*P* = 0.30
Ch1-2 cMD	0.588	0.028	0.590	0.025	0.586	0.026	*F* = 0.19	*P* = 0.83
Ch1-2 cAD	0.831	0.057	0.825	0.070	0.838	0.072	*F* = 0.416	*P* = 0.83
Ch4 (mm^3^)	0.001	0.056	0.011	0.071	−0.015	0.061	*F* = 1.79	*P* = 0.17 (uncorrected)
Ch4 FWf	0.408	0.074	0.399^b^	0.073	0.438^a,b^	0.058	*F* = 3.86, *t* = 2.04^a^, *t* = 2.78^b^	*P* = 0.04 *P* = 0.03 *P* = 0.01
Ch4 cMD	0.595	0.014	0.596	0.016	0.593	0.017	*F* = 0.41	*P* = 0.664
Ch4 cAD	0.842	0.059	**0**.**832**^b^	**0**.**045**	**0**.**863**^b^	**0**.**050**	***F* = 5.00, *t* = 3.05** ^b^	***P* = 0.04, *P* = 0.006**
PPN FWf	0.135	0.029	0.135	0.025	0.135	0.032	*F* = 0.01	*P* = 0.998
PPN cMD	0.596	0.002	0.596	0.003	0.597	0.002	*F* = 0.23	*P* = 0.798
PPN cAD	0.875	0.027	0.875	0.027	0.878	0.025	*F* = 0.20	*P* = 0.816

FW-corrected diffusivity data is multiplied by 1000. MoCA was missing at baseline in six PwP. PwP (CI) = people with PD with evidence of cognitive impairments (MoCA scores < 26); PwP (CN) = people with PD with no cognitive impairment (MoCA scores > 25). Bold indicates finding survives correction for age, sex and whole-brain structural metric.

Unless otherwise indicated FDR-corrected *P* values are reported. ANOVAs are corrected for number of diffusivity metrics within an ROI, and *t*-tests are corrected for number of *post hoc* comparisons made. Comparisons of volumetric measures are uncorrected (see ‘Methods’ section: Multiple comparisons). Negative volumes for Ch1-2 and Ch4 are due to normalization by total intracranial volume via ANCOVA. As such, normalized volumes have a mean of 0, and negative values indicate that volumes were smaller than expected given head size.

Significantly different to controls at *P* < 0.05.

Significantly different between the PD groups (with/without cognitive impairment).

There were no significant differences in the PPN in the according to disease group or cognitive status.

In controls, there were no significant correlations between structural metrics in the cholinergic nuclei and cognitive tasks that survived controls for age, sex and whole-brain structural metrics, as well as correction for multiple comparisons.

**Figure 2:**
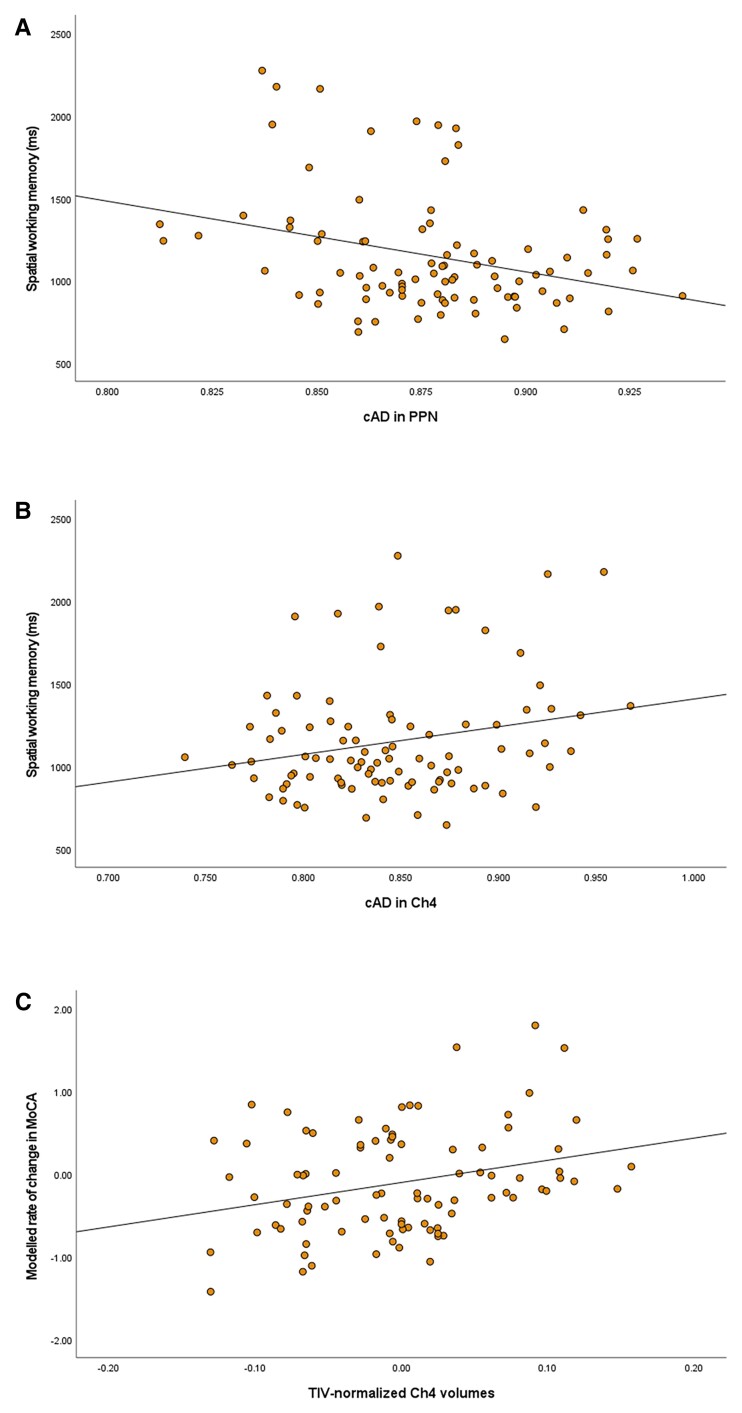
**Structural metrics and cognitive task performance.** (**A**) Scatterplot of cAD in PPN and reaction times on a spatial working memory task. (**B**) Scatterplot of cAD in Ch4 and reaction times on a spatial working memory task. (**C**) Modelled rate of change in global cognition (MoCA) and total intracranial volume-normalized volumes in Ch4. (Negative values indicate Ch4 volumes were smaller than predicted by TIVs).

Tables 3 and 5 report the FDR-corrected outcomes in people with PD. Of note, following correction for age, sex and whole-brain structure, metrics in the PPN were associated primarily with performance on attention tasks and spatial working memory, with elevated cAD being associated with faster reaction times on both task types (Fig. 2A).

**Table 3:** R values from baseline correlations between cognitive tasks and FW structural metrics in Ch1-2, Ch4 and PPN

	Ch1-2			Ch4			PPN		
	Fwf	cMD	cAD	FWf	cMD	cAD	FWf	cMD	cAD
**Global cognition**									
ȃMoCA	−0.224*	0.047	−0.040	**−0**.314**	0.095	−0.221*	0.048	−0.063	0.024
ȃMMSE	−0.172	−0.030	−0.162	−0.081	0.190	0.001	0.063	0.051	0.145
**Executive function**									
ȃFAS	−0.116	0.014	0.058	0.079	0.048	0.035	0.096	−0.020	−0.082
ȃAnimals	−0.279**	0.229*	0.073	−0.097	0.114	−0.065	0.039	0.126	−0.055
ȃOTS	−0.210*	0.116	0.078	−0.215*	−0.078	−0.057	0.099	0.051	0.050
**Memory**									
ȃPRM	−0.211*	0.148	0.081	−0.263**	0.083	−0.159	0.024	−0.013	0.029
ȃSRM	**−0**.290**	0.190*	0.087	−0.077	**0**.299**	0.172	−0.202	−0.084	−0.075
ȃPAL (TE)	0.057	−0.160	−0.127	0.183	−0.110	0.096	−0.044	−0.011	−0.064
ȃPAL (TT)	0.127	−0.171	−0.131	0.188*	**−0**.195*	0.027	−0.027	−0.073	−0.052
ȃPAL (MTS)	.210*	**−0**.268**	−0.146	0.249**	−0.054	0.108	−0.075	0.012	−0.104
**Attention**									
ȃSRT	0.105	0.064	0.050	0.225	0.051	0.152	−0.119	−0.036	−0.103
ȃCRT	0.266**	0.033	0.029	**0**.277**	0.010	0.168	0.028	0.002	**−0**.253*
ȃDV	0.167	0.067	0.079	0.053	−0.068	−0.030	0.053	0.121	−0.080
ȃCRT (Acc)	−0.030	0.077	−0.011	0.025	−0.192	−0.061	−0.100	−0.037	−0.067
ȃDV (Acc)	−0.114	0.110	0.083	−0.150	−0.092	−0.062	−0.008	−0.022	0.023
ȃPoA	0.194	0.066	0.031	0.268*	0.017	0.159	−0.004	0.065	−0.183
ȃPoA CV	0.181	0.092	0.080	0.162	0.128	0.154	0.087	0.221	0.001
ȃCog RT	0.282*	−0.089	−0.088	0.290*	−0.051	0.138	0.160	0.177	**−0**.233*
**Spatial working memory**									
ȃSWMOS	**0**.275**	−0.112	−0.059	**0**.364**	−0.063	0.179	−0.063	0.003	**−0**.228*
ȃSWMNS	0.125	−0.061	−0.098	**0**.389**	−0.083	0.229*	−0.064	−0.056	**−0**.310**
ȃSWM	0.196	0.000	−0.026	**0**.377**	−0.025	0.243**	−0.017	−0.025	**−0**.296**

Acc = accuracy as the percentage correct; Cog RT = cognitive reaction time; FAS = F-A-S test for phonemic verbal fluency; MTS = mean trials to success; SWM = SWM mean; SWMNS = SWM new stimulus; SWMOS = SWM original stimulus; TE = total errors; TT = total trials. Bold = partial correlation (additionally controlling for age, sex and whole-brain structural metric) significant at *P* < 0.05. *Bivariate correlation significant at *P* < 0.05 (FDR corrected for number of metrics within ROIs). **Bivariate correlation significant at *P* < 0.01 (corrected).

**Table 5:** R values from baseline correlations and beta weights for Structural Metric × Time interaction from linear mixed model of change in cognitive performance over 4.5 years

	TIV-normalized Ch1-2 volumes		TIV-normalized Ch4 volumes	
	R	β	R	β
**Global cognition**				
ȃMoCA	**0**.437**	4.07	0.212*	**3**.89*
ȃMMSE	0.246*	**5**.08**	0.116	2.32
**Executive function**				
ȃFAS	0.075	**30**.04**	0.001	**26**.41**
ȃAnimals	**0**.432**	4.42	0.260*	6.03
ȃOTS	**0**.393**	−0.60	0.282*	3.26
**Memory**				
ȃPRM	0.324**	3.80	0.231*	8.64
ȃSRM	**0**.370**	18.21	0.207*	13.85
ȃPAL (TE)	−0.344**	**−39**.03**	−0.234	**−25**.00*
ȃPAL (TT)	−0.346**	**−8**.07*	−0.219*	**−7**.55*
ȃPAL (MTS)	**−0**.427**	−0.61	−0.229*	−1.00
**Attention**				
ȃSRT	−0.213	−37.33	−0.137	−31.75
ȃCRT	−0.291**	−31.91	−0.173	−17.13
ȃDV	−0.302**	−53.02	−0.224	−3.03
ȃCRT (Acc)	**0**.317**	−0.52	0.187	0.15
ȃDV (Acc)	0.274*	**19**.72*	0.203	2.02
ȃPoA	**−0**.324**	−113.74	−0.204*	−66.13
ȃPoA CoV	**−0**.315**	0.95	−0.213*	−1.81
ȃCog RT	−0.172*	3.73	−0.074	3.88
**Spatial working memory**				
ȃSWMOS	−0.317**	−220.03	−0.245**	−360.99
ȃSWMNS	**−0**.392**	460.61	**−0**.351**	−99.30
ȃSWM	**−0**.393**	171.78	−0.318**	−209.34

Acc = accuracy as the percentage correct; Cog RT = cognitive reaction time; MTS = mean trials to success; SWM = SWM mean; SWMNS = SWM new stimulus; SWMOS = SWM original stimulus; TE = total errors; TT = total trials. Bold = significant at *P* < 0.05 (FDR corrected for number of diffusivity metrics compared). *Bivariate correlation significant at *P* < 0.05 (FDR corrected for number of metrics within ROIs). **Bivariate correlation significant at *P* < 0.01 (corrected).

cBF microstructure was associated with performance on a range of cognitive domains. However, in contrast to outcomes in the PPN, increased diffusivity or FWf in the cBF tended to be associated with ‘slower’ reaction times on timed tasks element (Fig. 2B).

### Do baseline structural metrics predict longitudinal change in cognitive performance?

Longitudinal changes in cognitive tasks and their relationship with baseline structural metrics in cholinergic nuclei were investigated with LMMs. Age, sex, baseline scores on tasks being modelled, baseline structural metric and performance at follow-up visits were entered into the model alongside the time × baseline structural metric interaction. Baseline Ch4 and Ch1-2 structural metrics were associated with progressive changes to global cognitive performance (Fig. 2C), executive function, memory and reaction times on attention tasks. (Statistical outcomes are reported in Tables 4 and 5). The PPN was not associated with performance changes on any cognitive task.

**Table 4:** Beta weights for Structural Metric × Time interaction from linear mixed model of change in cognitive performance over 4.5 years

	Ch1-2 × Time			Ch4 × Time			PPN × Time		
	FWf	cMD	cAD	FWf	cMD	cAD	FWf	cMD	cAD
**Global cognition**									
ȃMoCA	−1.14	6.70	1.76	−0.63	−2.31	0.32	0.56	3.30	0.63
ȃMMSE	−1.46	7.31	2.00	**−4**.01**	−1.29	−2.96	1.18	−34.84	−0.11
**Executive function**									
ȃFAS	**−8**.27*	−5.52	−9.90	−12.86	30.09	−6.93	−22.77	83.05	−9.90
ȃAnimals	−1.67	−0.42	−2.03	−2.83	30.33	1.69	−13.25	−156.36	4.45
ȃOTS	−3.06	1.60	−3.18	−1.71	19.70	−3.06	−13.74	−136.80	−2.79
**Memory**									
ȃPRM	−3.90	13.17	−2.59	1.67	22.01	11.04	−12.54	180.83	−4.36
ȃSRM	−6.33	−15.65	−6.09	−5.09	−60.35	−4.11	24.40	436.12	−11.52
ȃPAL (TE)	5.83	32.76	5.24	10.06	52.54	12.62	3.89	74.20	14.25
ȃPAL (TT)	1.48	11.31	3.45	3.51	21.68	6.97	3.10	21.43	0.16
ȃPAL (MTS)	0.22	1.16	0.35	0.37	1.20	0.52	0.43	5.93	0.07
**Attention**									
ȃSRT	**47**.17*	7.79	11.97	23.14	89.72	1.32	32.30	697.34	−136.44
ȃCRT	9.91	−42.53	−33.94	−19.02	−228.04	**−151**.23*	52.87	2077.36	78.64
ȃDV	7.05	−35.51	−36.76	**65**.75**	−114.18	−0.62	−44.37	−373.88	−143.11
ȃCRT (Acc)	0.89	−3.56	−0.15	−0.77	18.77	0.12	3.37	−19.13	−0.80
ȃDV (Acc)	−6.69	9.34	2.89	−3.54	44.94	8.26	−13.36	−81.83	−7.37
ȃPoA	56.40	91.83	10.18	74.21	−276.23	−99.63	113.10	1782.94	−263.02
ȃPoA CoV	−0.45	2.57	−1.20	2.85	−16.09	1.44	13.22	116.01	0.70
ȃCog RT	−47.72	71.85	1.35	−44.28	−423.41	−126.78	68.02	560.91	149.37
**Spatial working memory**									
ȃSWMOS	250.96	146.10	105.58	233.51	−1310.78	12.60	−5.62	2193.77	−260.09
ȃSWMNS	345.40	459.21	502.08	−164.97	−1171.07	−205.14	256.58	5128.06	−196.04
ȃSWM	303.07	242.74	323.87	1.63	−1301.89	−132.67	137.18	4012.36	−214.81

All models included control for age, sex, whole brain structure and baseline task performance. Acc = accuracy as the percentage correct; Cog RT = cognitive reaction time; MTS = mean trials to success; SWM = SWM mean; SWMNS = SWM new stimulus; SWMOS = SWM original stimulus; TE = total errors; TT = total trials. Bold = significant at *P* < 0.05 (FDR corrected for number of diffusivity metrics compared). *Bivariate correlation significant at *P* < 0.05 (FDR corrected for number of metrics within ROIs). **Bivariate correlation significant at *P* < 0.01 (corrected).

## Discussion

Free water imaging (both to capture FW content and to correct DTI metrics for the presence of FW) is emerging as an important tool for biomarker development in neurodegenerative diseases. When applied to the dopamine system, the technique has already been shown to distinguish people with PD from controls.^16–19,41–45^ However, it has not yet been applied to comprehensively characterize the cholinergic system in PD to our knowledge.

Using these methods, we also show that FWf in the Ch4 region of the cBF is greater in people with PD with current cognitive impairment compared to those with intact cognition and is correlated with baseline cognitive performance. On the other hand, and consistent with previous studies,^3–6^ volumetric measures of atrophy in this region could predict future, but not current, cognitive impairment. Ch1-2 volumes had a closer relationship with baseline cognitive performance and future cognitive impairment.

We also show that FW-corrected AD in the PPN was associated with faster baseline performance on cognitive tasks that required participants to make rapid choices between stimuli. Interestingly, the opposite pattern was observed in the cBF, where increased diffusivity was associated with ‘slower’ responses. The findings in the PPN were specific to baseline cognitive performance, suggesting that increased degeneration in this region has an impact on ability to behave flexibility during tasks requiring rapid responses, but that this is not reflective of the more global loss of cognitive function that occurs over time. We discuss this below in the context of our current understanding of PPN function and its role in PD.

Below, we discuss each of our findings in more detail.

### The PPN’s role in cognition

A substantial body of preclinical research now exists that has aimed to understand the PPN’s role in movement and cognition.^9,11–13^ Without this effort, it would be difficult to know how to interpret our current results in the human PPN.

Though the current study was not set up to specifically examine the role of the PPN in PD, the tasks employed allow us to interpret our findings alongside the preclinical literature. In awake rodents, non-cholinergic PPN neurons remain tonically active and do not respond to sensory inputs, while cholinergic PPN neurons show phasic short latency responses to sensory stimulation,^46^ implying they are involved in the rapid processing of sensory information. These studies, along with the PPNs descending connections to pontomedullary, cerebellar and spinal motor systems suggest strongly that a major function of the cholinergic PPN is participation in the generation of actions following initial processing of incoming sensory data. The tasks employed in the current study, in which rapid motor responses are required following presentation of attended visual stimuli, would therefore tap into PPN function well.

Recent findings indicate that the PPN plays an important role in behavioural flexibility via cholinergic output that inhibits the motor system through descending connections, and by inhibition of basal ganglia output.^9,11,13^ At baseline, we found faster responses on reaction time tasks in those with ‘greater’ PPN degeneration, which may reflect a loss of this inhibitory control. We also saw the same increase in reaction time on more complex tasks, including a spatial working memory task. Similar increases in reaction time have been reported for spatial working memory tasks in rats with PPN lesions, which came at the expense of the ability to react flexibly and adaptively.^47^ This loss of decision-making ability was also seen in the current paper, i.e. people with PD with greater cAD in the PPN took less time to consider choices between actions, therefore displaying faster cognitive reaction times. On the other hand, diffusivity increases in the cBF showed the opposite relationship, implying that while cBF degeneration resulted in slower task performance perhaps due to poorer cognitive ability, PPN degeneration had a more specific impact on flexible responding.

To extend on this point further, motor inhibition of the basal ganglia is achieved in part via PPN projections to striatal cholinergic interneurons, causing excitatory responses and, ultimately, inhibition of striatal spiny projection neurons.^10^ In addition, excitation of the subthalamic nucleus can occur via input from the PPN,^48^ which would theoretically increase activity in substantia nigra.^49^ Thus, PPN cholinergic activation of basal ganglia circuits would act to interrupt motor programs and decrease motor output.^11^

As such, our data suggests that in people with PD with PPN degeneration, inhibitory control is weakened, resulting in a failure to slow motor responses (hence faster reaction times) to accommodate the increased need to choose between competing motor responses. In other words, the processes required for behaving flexibility were employed less in those with more PPN degeneration.

It must be noted however that the tasks employed in the current study do not directly measure behavioural flexibility. Rather, the pattern of changes on tasks that require flexible responding allow us to interpret our data in the context of extensive preclinical literature.

Relatedly, the tasks used do not allow us to investigate the PPN’s role in reward-based learning via the ventral tegmental area and substantia nigra,^11^ but future work in this area should make use of the FW imaging tools we report. Suffice to say, it is increasingly necessary to investigate how basal ganglia activity responds to PD-related degeneration in PPN and its projections.

### Elevated FWf in the cBF in people with PD with evidence of cognitive impairment

In the cBF we were also able to extract volumetric data alongside microstructural data. We found that while there were no differences in cBF metrics between controls and people with PD as a whole, there was evidence of impaired microstructural integrity in the Ch4 region in people with PD with and without evidence of global cognitive impairment.^3–6^ It is likely that heterogeneity of cholinergic involvement in PD^50^ leads to non-significant differences when PD populations are considered as one homogenous group, particularly in early disease stages. This would additionally indicate that comparing metrics in the PPN between the full PD sample and controls may have yielded more significant results if we had separated the group by falls status or posture and gait symptoms. This will be the focus of future work, but the current findings support the growing recognition that structural imaging of the cholinergic systems can provide markers of cholinergic health that could stratify at-risk patients in clinical trials of cognitive interventions.

At baseline, FWf in Ch4 was also correlated with baseline cognitive performance across a range of cognitive tasks, but volumetric measurements in this region were more likely to be predictive of future cognitive decline. Both findings are consistent with recent multimodal imaging studies with longitudinal follow-up in PD.^4^ These findings imply that FWf and volume measures provide complimentary information about the progressive changes in cholinergic nuclei in PD. Microstructural changes occur earlier and may better reflect ongoing inflammatory and neurodegenerative processes that are acting to impair cognitive abilities, while volume changes due to cell loss may better reflect the likelihood that cognitive impairment will progress over time. This is important because a neuroimaging biomarker of the cholinergic system will be most successful if it is sensitive to dynamic changes to current and future degenerative processes.

We also found that people with PD without cognitive impairment had larger volumes than those with cognitive impairment and controls in Ch1-2. This potentially reflects a mechanism by which cognitive function is maintained in some PD and is consistent with a recent study finding greater vesicular acetylcholine levels in the hippocampus (which receives cholinergic projections from Ch1-2) in people with PD with normal cognition, compared to healthy controls or people with cognitive impairment.^51^ This would further imply that differences in Ch1-2 volumes in people with PD with/without cognitive impairment, at least at early disease stages, are not disease related, which is consistent with our finding that these differences do not survive correction for age.

There are limitations related to the imaging methods used here. While the FW model can be estimated from single-shell diffusion MRI data, it requires some regularizations and does not address limitations related to crossing fibres. Alternative diffusion MRI acquisitions (such as multi-shell) and analysis methods must be employed to ensure the analysis of the FW-related metrics becomes more robust and accurate.

In addition, there are differences in structural organisation and anatomical location between the PPN and cBF that may result in different contributions from white matter and CSF contamination, respectively. This means we cannot be sure that diffusivity metrics are representing the same pathology with the same sensitivity in both regions. That said, free water imaging in the substantia nigra is a highly promising progression biomarker for PD,^52^ and work is ongoing to understand how FW and DTI metrics represent brain pathology more widely. Of note, high-field imaging studies suggest there may be a specificity for FW metrics for neuroinflammatory processes,^53^ while DTI metrics may be differently responsive to accumulation of pathological protein aggregates and inflammatory immune activation.^54^ Of particular relevance for the current paper, high-field imaging has also revealed changes in DTI metrics in regions that develop α-synuclein pathology and immune activation in PD mouse models that precede the onset of symptoms.^55^

### The link with postural instability, gait impairment and falls

The link between postural instability/gait impairment/falls and attention is now well recognized.^23,56^ Previous data suggest that the degree to which dual task interference worsened gait in people with PD is correlated with PPN structural connectivity.^57^ In addition, we have previously showed that PPN diffusivity metrics and Ch4 volumes could predict which people with PD were at risk for postural instability and gait deficits.^8,26^ Taken together, these findings indicate that the changes in Ch4 and PPN that lead to impaired behavioural flexibility and attention also led to a loss of ability to respond adaptively when navigating natural environments, therefore leading to posture and gait deficits and falls. It is now necessary to develop a more detailed understanding of these links if we are to design effective interventions that target the cholinergic system.

## Conclusion

We reveal that changes in cholinergic nuclei can be detected in people with PD that may reflect disease heterogeneity. Structural changes in the cBF may be relevant for cognitive impairment across multiple cognitive domains. Degeneration in the PPN may be associated with tasks that depend on rapid updating of actions in response to changing environmental contingencies, consistent with the animal literature. Recent data indicate that the PPN plays a role in regulating basal ganglia activity and could be targeted to improve nigrostriatal dopamine signalling.^58^ The current study indicates that FWf and FW-corrected DTI could be a useful to investigate the role of the PPN in PD in the human, so that strategies for targeting the PPN can be rationally designed in the context of disease.
